# Neurofilament light chain as a marker for neuronal damage: integrating in vitro studies and clinical findings in patients with oxaliplatin-induced neuropathy

**DOI:** 10.1007/s00280-025-04773-w

**Published:** 2025-04-10

**Authors:** Nina Lykkegaard Gehr, Christina Mortensen, Tore B. Stage, Malene Roland Vils Pedersen, Søren Rafael Rafaelsen, Jonna Skov Madsen, Dorte Aalund Olsen, Signe Timm, Lars Henrik Jensen, Torben Frøstrup Hansen, Nanna Brix Finnerup, Lise Ventzel

**Affiliations:** 1 Danish Pain Research Center, Department of Clinical Medicine, Aarhus University, Aarhus, Denmark; 2 Department of Oncology, University Hospital of Southern Denmark, Vejle, Denmark; 3 Clinical Pharmacology, Pharmacy, and Environmental Medicine, Department of Public Health, University of Southern Denmark, Odense, Denmark; 4 Department of Regional Health Research, Faculty of Health Sciences, University of Southern Denmark, Vejle, Denmark; 5 Department of Clinical Biochemistry and Immunology, University Hospital of Southern Denmark, Vejle, Denmark; 6 Department of Radiology, University Hospital of Southern Denmark, Vejle, Denmark; 7 Colorectal Cancer Center South, Vejle Hospital, University Hospital of Southern Denmark, Vejle, Denmark

**Keywords:** Chemotherapy-induced peripheral neuropathy, Neurofilament light chain, Colorectal cancer, Biomarker, Sensory neurons

## Abstract

**Purpose:**

Oxaliplatin-induced peripheral neuropathy (OIPN) is a chronic, debilitating late effect following oxaliplatin treatment. Neurofilament light chain (NfL) is a structural protein found in nerve axons that was investigated upon oxaliplatin exposure in vitro and in vivo correlated to symptoms of OIPN in colorectal cancer patients receiving oxaliplatin.

**Methods:**

Human sensory neurons, derived from induced pluripotent stem cells, were exposed to clinically relevant concentrations of oxaliplatin in vitro, with NfL concentrations measured in the cell medium. The prospective clinical study included patients with colorectal cancer undergoing chemotherapy therapy with or without oxaliplatin. Possible OIPN was defined as bilateral presence of numbness and/or presence of pricking sensations in the feet documented in an interview at the time of blood sampling prior to, 3, and 6 months after initiating treatment.

**Results:**

Oxaliplatin exposure led to a dose-dependent NfL increase *in vitro.* In the clinical cohort of 30 patients (18 in the oxaliplatin group), NfL levels rose at 3 and 6 months compared to controls. NfL level changes correlated to OIPN symptoms at the 6-month timepoint (rho 0.81, *p* < 0.001). However, the interindividual variation was substantial, and most patients showed only a minor increase in NfL.

**Conclusion:**

Both in vitro and clinical data indicate that oxaliplatin exposure results in elevated NfL levels. Further prospective studies are needed to evaluate NfL as an early biomarker for OIPN, specifically focusing on the timing of blood sampling during chemotherapy treatment to enable the timely reduction of oxaliplatin.

**Supplementary Information:**

The online version contains supplementary material available at 10.1007/s00280-025-04773-w.

## Introduction

Oxaliplatin-induced peripheral neuropathy (OIPN) is a well-known consequence of cancer treatment with the chemotherapeutic agent oxaliplatin. Chronic OIPN afflicts more than half of oxaliplatin-treated cancer patients, with 20% experiencing neuropathic pain [1, 2]. This dose-dependent peripheral neuropathy is often characterized by a delayed development known as “coasting”. Patients commonly report persistent para- and dysesthesia, as well as neuropathic pain primarily in a stocking and glove distribution [3]. Chronic OIPN, including neuropathic pain, significantly impairs the quality of life for cancer patients [4, 5]. It is associated with functional loss, reduced proprioception, and patients with CIPN report psychological symptoms such as anxiety, depression, and sleep disturbances more often, all of which place a substantial burden on the healthcare system [6–8]. Chronic OIPN remains without an effective treatment, as several clinical trials testing various drugs have yet to yield a medication that can relieve or prevent this serious side effect [9]. Duloxetine is the only drug shown to have some effect on painful OIPN [10]. The only way to avoid OIPN is to reduce or cease oxaliplatin in a timely manner. Still, due to the coasting phenomenon, the severity of patient-reported symptoms is detected with a latency. Therefore, there is a need for an early biomarker of OIPN.

Neurofilament light chain (NfL) is a structural protein found abundantly in axonal neurons. Upon neuronal damage, NfL is released and is detectable in cell culture media and animal and human peripheral blood using ultra-sensitive assays [11–13]. NfL has proven to be a useful biomarker in various central nervous system (CNS) conditions, including multiple sclerosis, traumatic brain injury, and Alzheimer’s [14]. Several studies have found a correlation between increased circulating NfL and symptoms of chemotherapy-induced neuropathy caused by other types of neurotoxic chemotherapy, such as paclitaxel [15–19]. Based on prevailing theories of OIPN, including damage to peripheral nerves and dorsal root ganglia, NfL has the potential to be a relevant biomarker of OIPN [20]. The aim of this study was to explore NfL levels upon oxaliplatin exposure on a cellular level in vitro. Furthermore, we aimed to study NfL levels in a clinical setting in patients with colorectal cancer treated with oxaliplatin and explore the relation between NfL and symptoms of OIPN.

## Materials and methods

### In-vitro study

#### Sensory neuron differentiation

Two human iPSC lines from healthy donors were obtained commercially (A18945, ThermoFisher, Roskilde, Denmark, hpscreg.eu/cell-line/TMOi001-A; WTC-11, Gladstone Institute of Cardiovascular Disease, University of California San Francisco, hpscreg.eu/cell-line/UCSFi001-A). The iPSCs were maintained, passaged, and differentiated into sensory neurons as previously described and confirmed in detail [21]. Briefly, iPSCs were maintained in mTeSR1 medium (85850, StemCell Technologies, Vancouver, BC, Canada) on Matrigel-coated 6-well plates (354277, Corning, NY, USA) at a minimum density of 50,000 cells/cm^2^. The medium was changed daily, and iPSCs were routinely passaged at ∼70–80% confluency using Accutase (00455556, ThermoFisher). The differentiation was performed using 5 small molecule inhibitors for 12 days, followed by maturation with four neurotrophic growth factors and ascorbic acid (A4403, Sigma-Aldrich, Saint Louis, MO, USA) for an additional 23–33 days. On day 12, immature sensory neurons were seeded as single cells at a density of 150,000 cells/cm^2^ on culture plates coated with poly-L-ornithine hydrobromide (20 µg/mL, P3655, Sigma-Aldrich), laminin (10 µg/mL, 23017015, ThermoFisher), and fibronectin (2 µg/mL, F1141, Sigma-Aldrich). On day 14, non-neuronal cells were removed using Mitomycin-C (1 µg/mL, M4287, Sigma-Aldrich) for 2 hours. On day 16, all medium was replaced; afterwards, 50% of the medium was changed every 3–4 days. The mature sensory neurons were used for experiments between days 35–45.

#### Chemotherapy exposure

Oxaliplatin (O9512, Sigma-Aldrich) was dissolved and serially diluted in sterile water. The final concentration of sterile water was maintained at 0.2% for all conditions, and the same concentration of sterile water was included as a control. The concentrations of oxaliplatin were chosen based on the maximum plasma concentrations observed in clinical pharmacokinetic studies and adjusted to account for the inability to carry out repeated oxaliplatin exposure [22]. After 96 h of exposure to oxaliplatin, the medium was collected, and the respective iPSC-derived sensory neurons (iPSC-SNs) were lysed using radioimmunoprecipitation assay buffer (89900, ThermoFisher).

#### Immunolabeling

Human induced pluripotent stem cell-derived sensory neurons (iPSC-SNs) were fixed with 4% paraformaldehyde for 10 min, washed twice with phosphate-buffered saline (PBS, D8662, Sigma-Aldrich), and subsequently permeabilized with 0.25% Triton X-100 for 15 min. An unspecific binding was subsequently blocked using 1% bovine serum albumin (A9418, Sigma-Aldrich) for one hour. iPSC-SNs were labeled with NFL monoclonal antibody (1:100, MA5-14981, ThermoFisher) overnight at 4 °C. The following day, iPSC-SNs were labeled with Alexa Fluor 488-conjugated anti-rabbit (1:400, A11008, ThermoFisher) for 1 h at room temperature. Images were acquired using ImageXpress Pico Automated Imaging System with the 10X objective (Molecular Devices, San Jose, CA, USA).

#### Analysis of cell data

iPSC-SN experiments were performed for two individual donors with three technical replicates per condition. NfL levels were adjusted for protein concentrations, and fold changes in NfL levels were calculated relative to the mean value of the vehicle control for each individual differentiation.

### Clinical study

From 2019 to 2021, patients diagnosed with colon cancer seen in the Department of Oncology, Vejle Hospital, and University Hospital of Southern Region of Denmark were invited to participate in the study. Eligible participants were adult patients aged 18–90 with a diagnosis of colorectal cancer stage III or IV. Patients followed treatment regimens according to clinical guidelines, which led to the formation of two groups based on whether their regimen included oxaliplatin. An oxaliplatin regimen comprised a minimum of four planned treatment cycles of oxaliplatin (130 mg /m^2^) in combination with capecitabine (2000 mg/m^2^) every three weeks. The non-oxaliplatin treatment regimens comprised eight planned treatment cycles of capecitabine (2000 mg/m^2^) every three weeks or a treatment regimen including folinic acid, fluorouracil, and irinotecan every two weeks. Exclusion criteria for both groups were a history of oxaliplatin treatment, lumbar radiculopathy, lower extremity peripheral neuropathy, or peripheral vascular disease. Patients were evaluated prior to treatment, after 3 months, and after 6 months. These visits coincided with the patient’s oncological treatment schedule, with the 3-month evaluation occurring approximately at the 5th treatment cycle and the 6-month evaluation around the 8th cycle. At each visit, OIPN evaluation and blood sampling were carried out.

#### OIPN evaluation

An oncologist evaluated the symptoms of OIPN in an interview, including dichotomized presence of the following symptoms in each lower extremity: numbness, pricking sensation, pain, muscle weakness, and ataxia. Each positive answer generated one point resulting in a combined score of both extremities ranging from 0 to 10. We defined possible OIPN as the bilateral presence of numbness and/or presence of pricking sensations in the feet as previously done [1, 3], correlating to the score of minimum 2 on the transformed possible OIPN grading scale.

Information on treatment modality accumulated oxaliplatin dosage, the reason for dose reduction and premature cessation, and certain comorbidities were obtained from patient records from the oncological department during the treatment period.

#### Blood sampling

Blood samples (10 mL) were collected by venous puncture and drawn into serum tubes according to standard procedure. After allowing the blood to coagulate for 30 min, the samples were centrifuged at 2,000 g for 10 min. The supernatant was then stored at -80 °C until use.

#### NfL analysis

NfL measurements of iPSC-SN medium and patient serum were conducted at the Department of Biochemistry and Immunology, University Hospital of Southern Denmark, Vejle, Denmark. Measurements were performed blinded to experimental and clinical data using a commercially available NfL assay on the Single molecule array (Simoa^®^) HD-X Analyzer (Quanterix, Billerica, MA, USA). iPSC-SN medium samples were diluted 100-fold in buffer included in the assay kit and analyzed in duplicates. Serum samples were diluted four-fold in the buffer and analyzed as single determinations. Quality control was performed using two controls prepared from commercially available control material provided by the manufacturer in addition to an in-house prepared serum pool. The quality controls were included in each run for evaluating and monitoring assay performance. The analytical coefficients of variation were below 13%.

#### Study approval and statement of human and animal rights

The protocol was approved by the Ethical Committee of the Southern Region of Denmark (S-20190048 approved Aug 5th, 2019). Patients provided written and informed consent before enrolment in the study. The study is non-interventional and collected data was anonymized.

#### Statistics

Descriptive statistics were used to describe patient demographics in the two groups. Numerical data were analyzed using Wilcoxon rank-sum test due to non-normal distribution. Linear mixed model analysis was used to estimate the predicted levels of NfL and possible OIPN score in the two groups and visualized using margin plots. Relevant adjustments were determined using directed acyclic graphs (DAGs), and age and diabetes were adjusted for [23]. Post-estimation Wald test was used to determine statistically significant different levels between repeated measurements. The mixed model took missing values into account.

Linear regression evaluated the association between accumulated oxaliplatin dose and NfL and possible OIPN score at different time points, including only complete cases. The correlation between NfL and possible OIPN score was calculated using Spearman correlation and linear regression. Statistical significance level was determined to be α = 0.05. Data analysis was carried out in STATA/SE version 18.

## Results

### In-vitro results

**Fig. 1:**
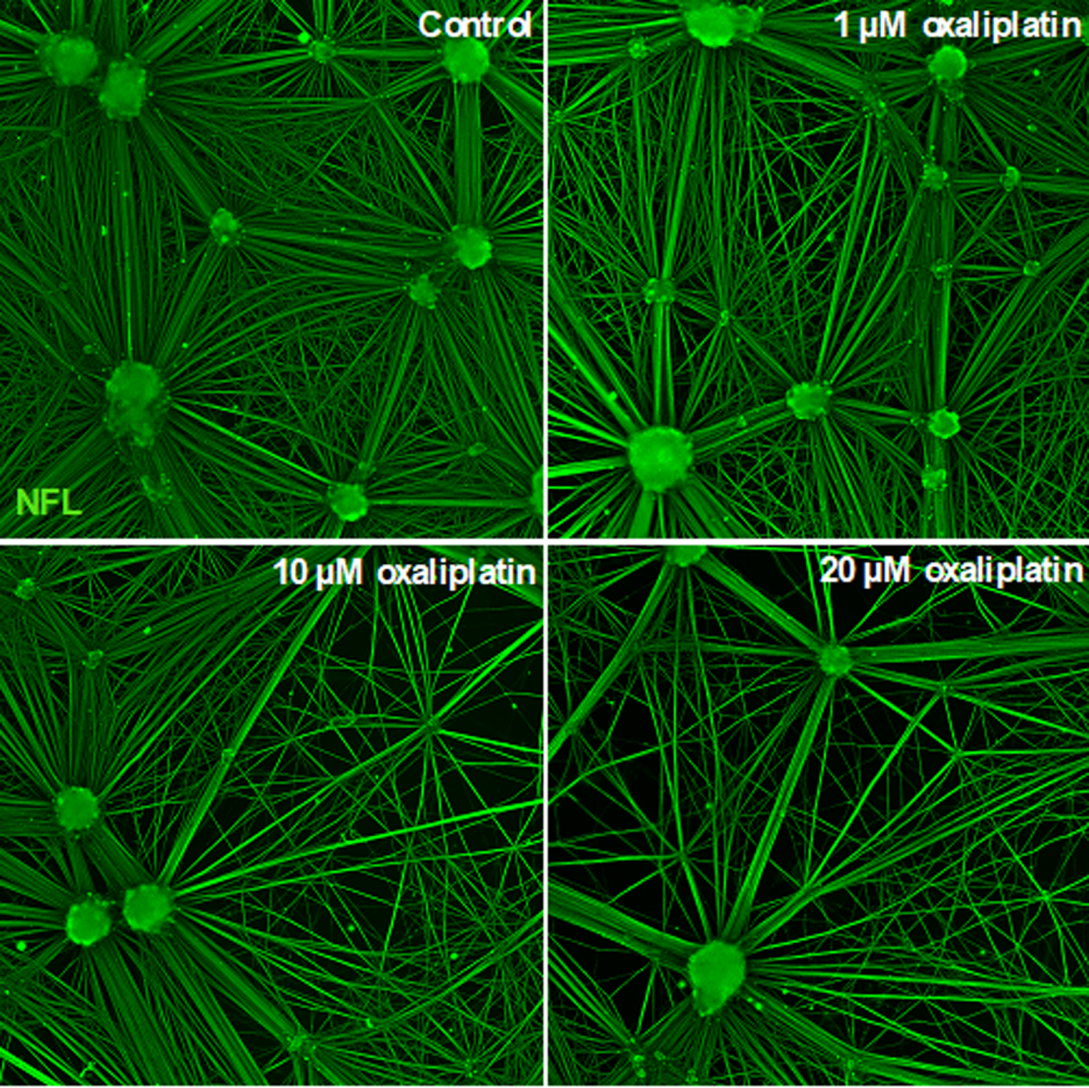
Oxaliplatin caused axonal damage to iPSC-derived sensory neurons in a concentration-dependent manner. Cells were exposed to control (sterile water) or 1, 10, or 20 µM oxaliplatin for 96 h. Cells were fixed, permeabilized, and labeled with NfL. Images were acquired using ImageXpress Pico Automated Imaging System with the 10X objective. Representative images are shown for the indicated concentrations. The experiment was performed with the iPSC donor A18945. iPSC, induced pluripotent stem cell; NfL, neurofilament light chain

**Fig. 2:**
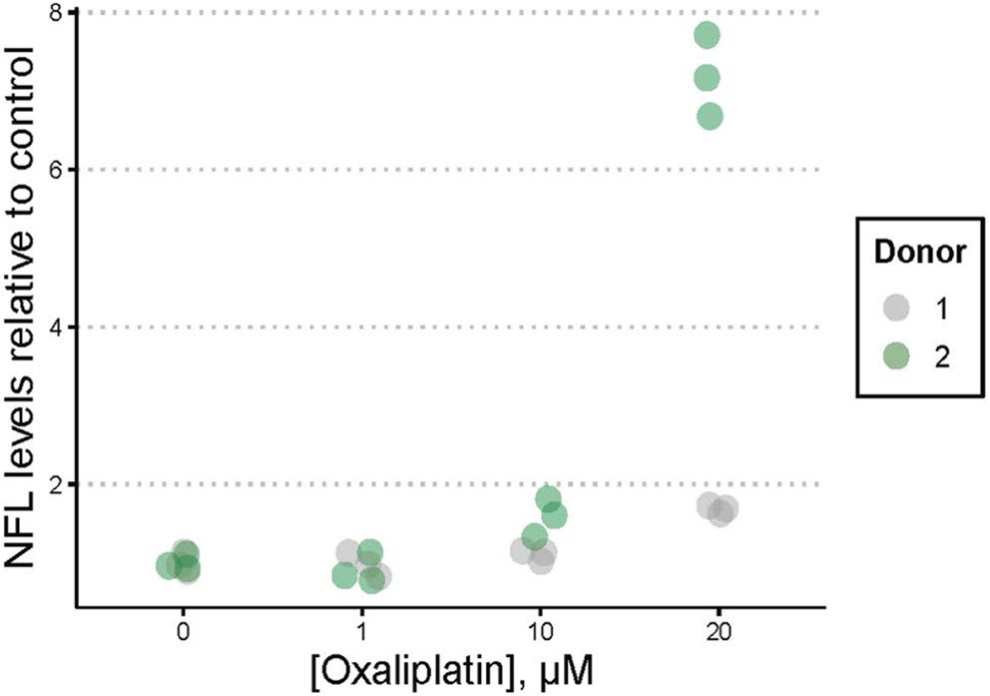
Neurofilament light chain (NfL) concentrations in medium from induced pluripotent stem cell-derived sensory neurons following oxaliplatin exposure. NfL concentrations were measured in the medium collected from induced pluripotent stem cell-derived sensory neurons after 96 h of exposure to oxaliplatin, and NfL concentration were subsequently measured using single-molecule array. The experiment included iPSC donors A18945 (donor 1) and WTC-11 (donor 2)

Oxaliplatin caused concentration-dependent axonal damage to iPSC-SNs, seen morphologically as a reduction of the otherwise highly organized neuronal network (control) (Fig. 1). In line with the observed axonal damage, NfL concentrations were substantially elevated at the highest concentrations of 10 and 20 µM oxaliplatin, although with great variation between donors (Fig. 2). Absolute NfL levels in media and protein concentrations used for normalization are shown in Table S1.

### Clinical results

**Table 1:** Baseline characteristics. BMI: body mass index, IQR: interquartile range

	Non-oxaliplatin	Oxaliplatin
N	12	18
Age median (IQR)	69 (60–72)	60 (54–64)
BMI median (IQR)	28.9 (22.2–40.2)	27.8 (19.9–37.9)
Sex, male n (%)	7 (58.3)	9 (50.0)
Cancer stage n (%)		
III	6 (50.0)	10 (55.6)
IV	6 (50.0)	8 (44.4)
Diabetes n (%)	1 (8.3)	2 (11.1)
Alcohol consumption n (%)		
less than 10 units/week	5 (41.7)	7 (38.9)
more than10 units/week	1 (8.3)	5 (27.8)
Smoking n (%)		
never	7 (58.3)	9 (50.0)
former	3 (25.0)	6 (33.3)
current	0 (0.0)	3 (16.7)
No. cycles of oxaliplatin median (IQR)	-	4 (3–6)
No. cycles oxaliplatin in 100% dose median (IQR)	-	2 (1–3)
Dose oxaliplatin median mg/m^2^ (IQR)	-	513 (408–873)

**Fig. 3:**
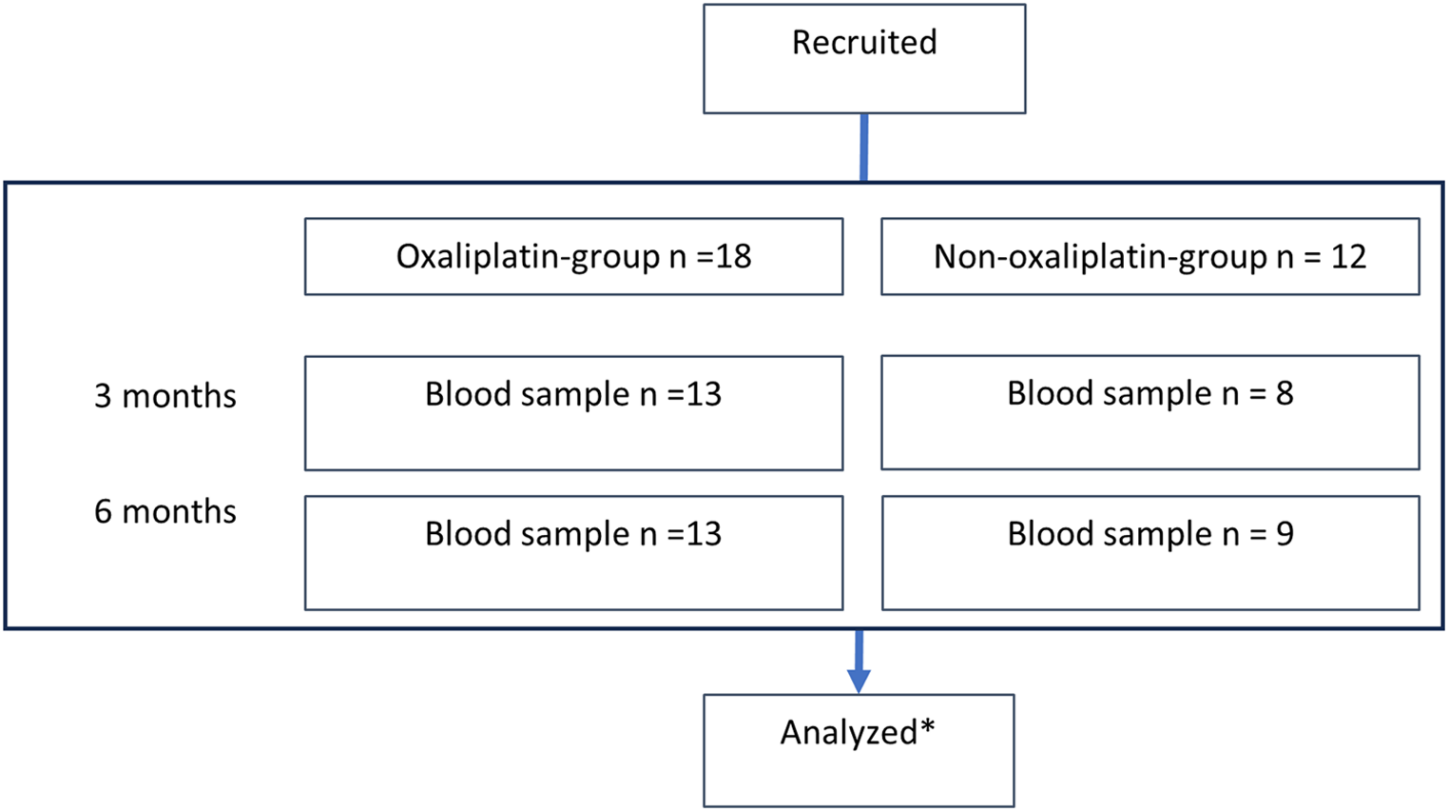
Flowchart, including available data at 3-month and 6-month timepoints. *All patients are included in the mixed analysis

A total of 30 patients were included. Eighteen patients received oxaliplatin as part of their chemotherapeutic regimen, and 12 patients received the non-oxaliplatin regime. Several incomplete blood samples and OIPN evaluations were seen at the 3- and 6-months evaluations. The study was a pragmatic study embedded in a clinical routine which caused several missing samples and evaluations for logistic reasons and therefore random (Fig. 3). However, all patients had at least two blood samples and OIPN evaluations. Patients in the oxaliplatin group were slightly younger than those not receiving oxaliplatin (Table 1).

#### Chemotherapy treatment

Patients in the oxaliplatin group underwent treatment with oxaliplatin and capecitabine (CAPOX) either as an adjuvant or neoadjuvant regimen. In the non-oxaliplatin group, seven patients received capecitabine alone, and five patients received folinic acid, fluorouracil, and irinotecan. Depending on the severity of adverse effects, the dose was either reduced or prematurely stopped. If the dose was reduced, the initial reduction was to a dose of 75% and subsequently 50% of the standard dose if there was persistent adverse effect despite the dose reduction. If patients experienced persistent symptoms of neuropathy on the treatment day, adjustments were made. The type of adjustment depended on the severity and progression rate, but all patients experienced at least persistent neuropathy symptoms. The median number of treatment cycles with oxaliplatin was four (IQR: 3–6), and the median number of cycles with an oxaliplatin dose of 100% was two (IQR:1–3). Oxaliplatin dose was reduced in 44% (8/18) of the treatment regimens due to neuropathy symptoms and in 11% (2/18) due to other adverse events. The dose reduction occurred after a median of two cycles (IQR:1–3). Prematurely cessation of oxaliplatin due to neuropathy symptoms occurred in 56% (10/18) of cases after a median of four cycles (IQR:3–6). Of the 10 cases, five had undergone dose reduction before the oxaliplatin treatment was prematurely stopped. 16.7% (3/18) solely due to other adverse events or progression. At the 3-month timepoint (5th cycle) and the 6-month timepoint (8th cycle), 44% (8/18) and 11% (2/18) respectively were in active oncological treatment. Cumulative oxaliplatin dose was 513 mg/m^2^ (IQR: 408–873 mg/m^2^) (Table 1).

#### Serum NfL levels

**Fig. 4:**
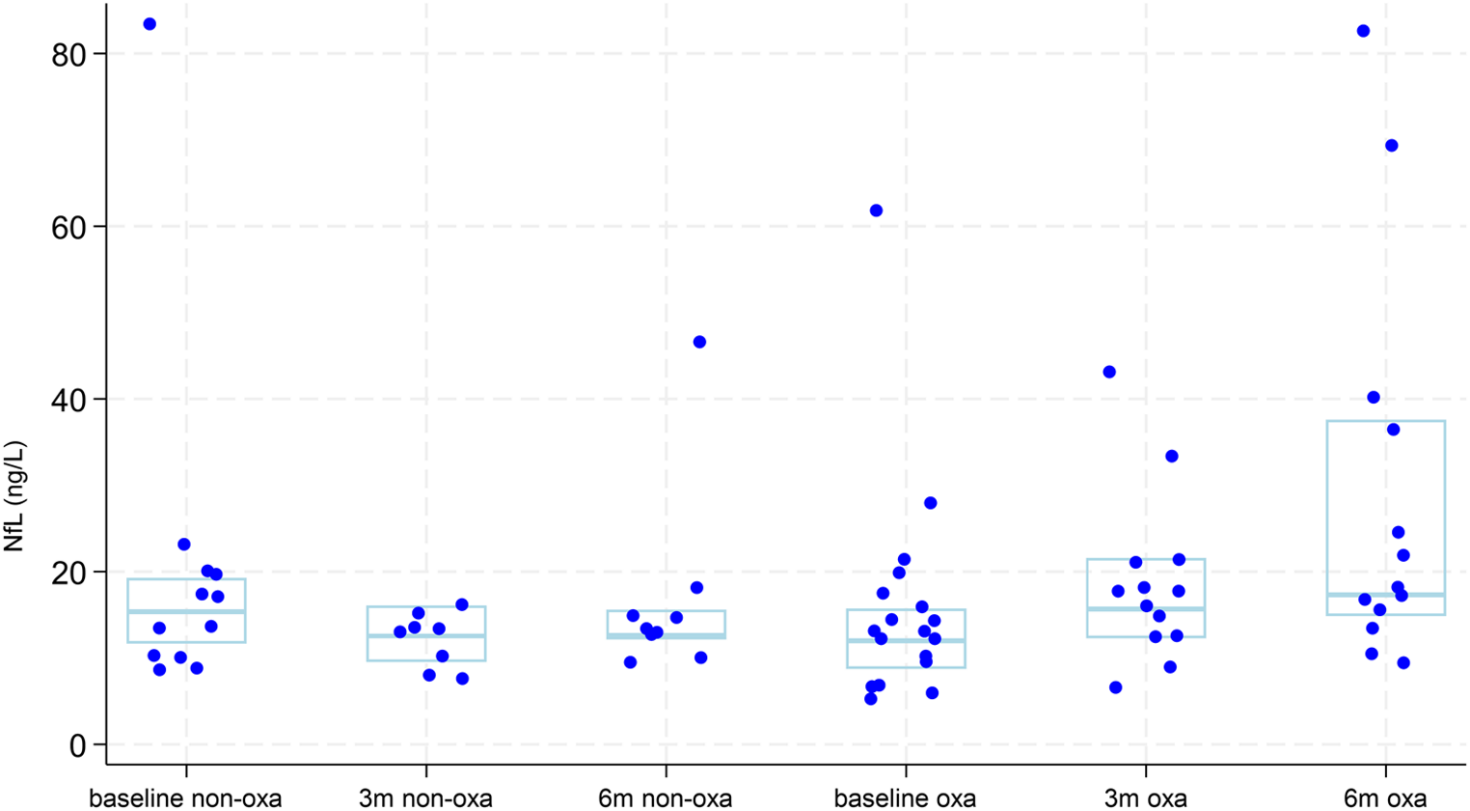
Dot plot of NfL levels at the 3 timepoints for measurement (baseline, 3 months and 6 months) for patients who received oxaliplatin (oxa) and patients who did not receive oxaliplatin (non-oxa). Light blue box represents median and interquartile ranges. NfL: Neurofilament light chain protein

Overall median baseline NfL concentration was 13.1 ng/L (IQR: 10.2–18.5), which falls within the reference range for the corresponding age group [24]. The median NfL concentration was 12.0 ng/L (IQR: 8.7–15.7) in the oxaliplatin group and 15.4 ng/L (IQR: 11.7–19.3) in the non-oxaliplatin group, with no significant difference (*p* = 0.13) (Fig. 4; Table 2). Three baseline outliers with NfL concentrations exceeding 30 ng/L were identified, with no medical explanation found in the patient records. However, the respective NfL levels at 3 months were within the expected range. Due to a substantial amount of interindividual variance, changes in NfL concentrations at 3 months (Δ3NfL) and 6 months (Δ6NfL) from baseline were calculated. The Δ3NfL was different between the oxaliplatin and non-oxaliplatin groups (median 3.6 ng/L (IQR: -0.3-7.4) vs. -2.2 ng/L (IQR: -3.3- -1.3), *p* = 0.02). Similarly, Δ6NfL showed a significant difference (median 11.6 ng/L (IQR: 3.6–26.0) vs. -3.6 ng/L (IQR: -7.1- -0.6), *p* = 0.001) (Table 2).

#### OIPN evaluation

Two patients had unilateral symptoms at baseline, and no new unilateral symptoms were reported. Possible OIPN was reported by 33% (3/9 patients, 9 missing) at the 3-month timepoint and 70% (7/9, 9 missing) after 6 months in the oxaliplatin group. Despite a substantial number of missing OIPN evaluations, a significant difference in the median possible OIPN score is seen at the 6-month timepoint (Table 2).

**Table 2:** Median measurement of neurofilament light chain levels (NfL) and DeltaNfL levels. Measurements of the possible OIPN score at different time points, including no. of observations. Wilcoxon signed rank test of measurements of the two groups. (IQR: interquartile range)

	No.	Oxaliplatin, median (IQR)	No.	Non -oxaliplatin, median (IQR)	Wilcoxon signed rank test (*p*)
NfL (ng/L)					
Baseline	18	12.0 (8.7–15.7)	12	15.4 (11.7–19.3)	0.13
3 months	13	15.7 (12.3–21.6)	8	12.5 (9.6–16.1)	0.27
6 months	13	17.3 (14.9–37.6)	9	12.6 (12.1–15.6)	0.21
Δ NfL (ng/L)					
Δ3NfL	13	3.6 (-0.3-7.4)	8	-2.2 (-3.3 - -1.3)	0.02
Δ6NfL	13	11.6 (3.6–26.0)	9	-3.6 (-7.1 -0.6)	< 0.01
Possible OIPN score (0–10)					
Baseline	18	0 (0)	12	0 (0)	
3 months	9	0 (0)	7	0 (0)	0.30
6 months	10	3.5 (0–4)	9	0 (0)	< 0.01

**Fig. 5:**
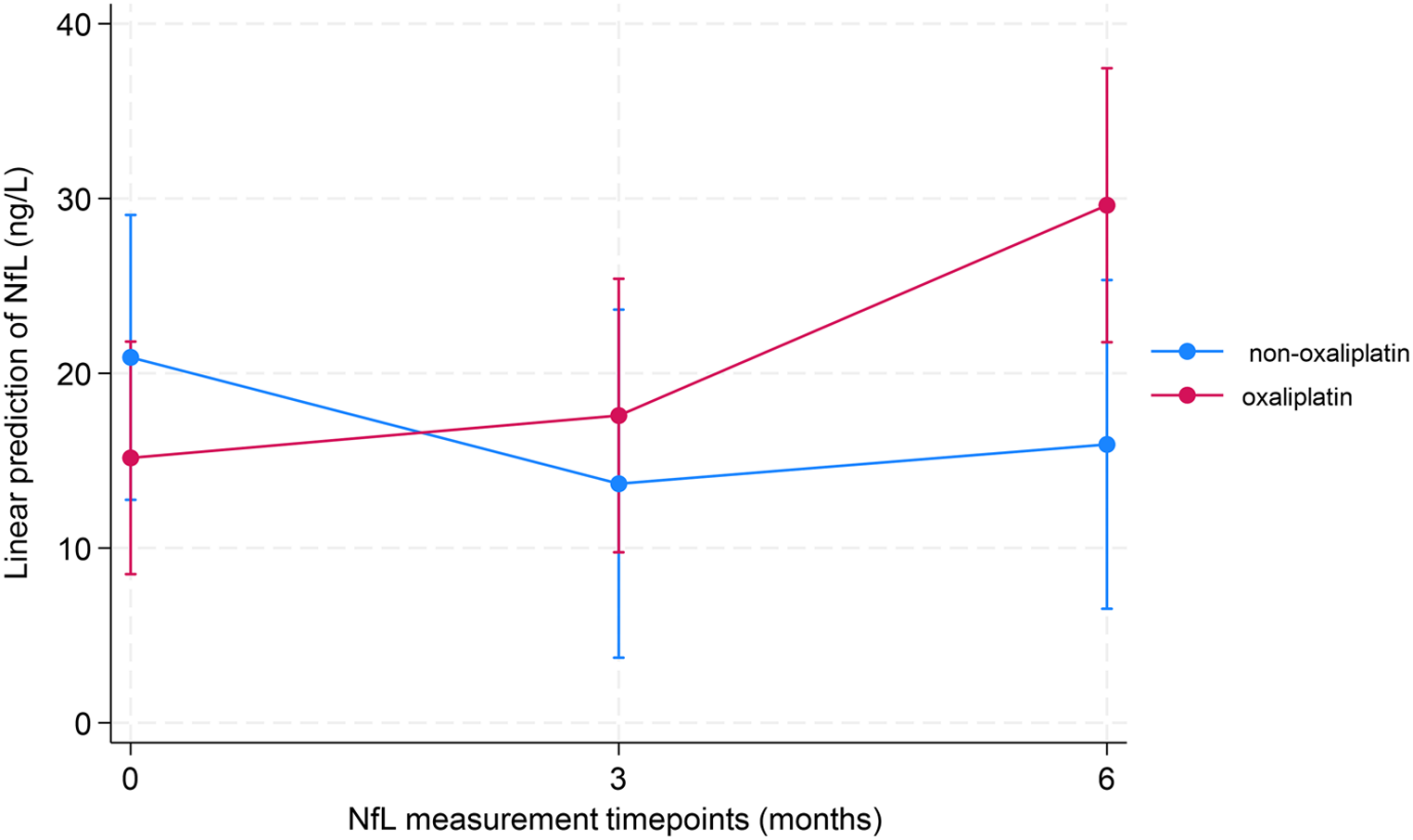
Predicted NfL values at 3 timepoints in the two groups based on linear mixed model adjusted for age and diabetes visualized with 95% CIs

Taking the missing values of NfL and possible OIPN score into account (Table 2), a linear mixed model was performed. The predicted difference in absolute NfL levels between the oxaliplatin group and the non-oxaliplatin groups at 3 months was 9.48 ng/L (95% CI: -6.50–25.46, *p* = 0.25) and at 6 months 18.02 ng/L (95% CI:2.41–33.63, *p* = 0.02). When adjusting for age and diabetes, the predicted differences in NfL levels between the two groups at 3 and 6 months were 9.65 ng/L (95% CI: 6.28–25.59, *p* = 0.23) and 19.44 ng/L (95% CI: 3.79–35.09, *p* = 0.02), respectively. Postestimation Wald test showed a significant difference between the predicted NfL differences at 3 and 6 months (*p* = 0.05). The predictive coefficients and belonging margins are illustrated in the margins plot in Fig. 5.

**Fig. 6:**
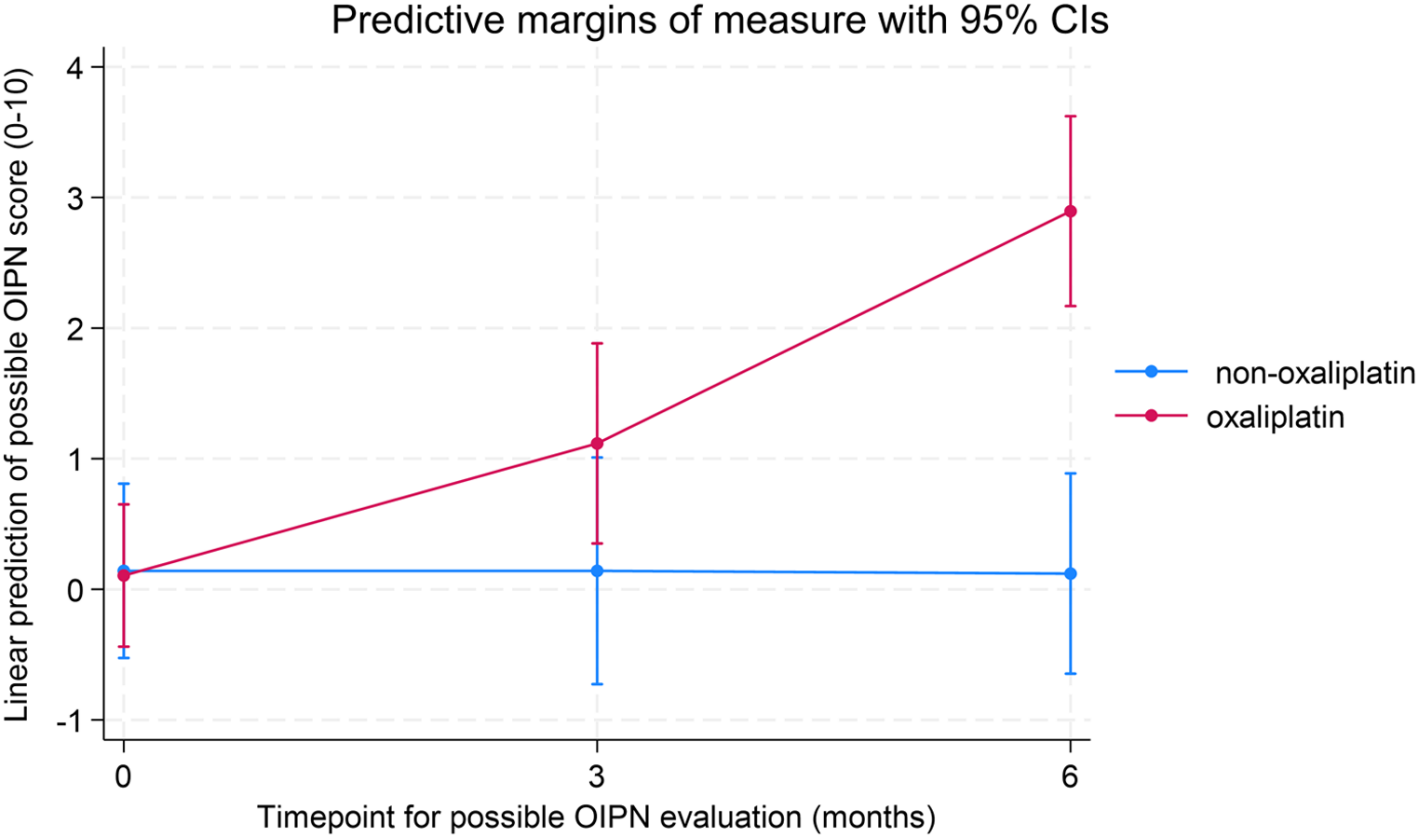
Linear mixed model of predicted possible OIPN scores at 3 and 6 months adjusted for age and diabetes visualized with 95% CIs margins plot

The linear mixed analysis of the predicted difference in possible OIPN score between the two groups at 3 months was 1.05 (95% CI: -0.33- 2.44, *p* = 0.14) and 2.83 (95% CI: 1.53–4.13, *p* < 0.01) at 6-month timepoint. Adjusting for diabetes and age did very little to the coefficients. 3 months adjusted coefficient is 1.01 (95% CI: -0.38–2.40, *p* = 0.15), and at 6 months, 2.81 (95% CI: 1.51–4.11, *p* < 0.01 Postestimation Wald test of no difference between the two predicted possible neuropathy score differences at 3 and 6 months renders a p-value of < 0.01, suggesting a significant difference between the two predicted possible OIPN score differences. The predictive coefficients and margins are illustrated in the margins plot in Fig. 6.

#### Correlation of possible OIPN score and NfL levels

**Fig. 7:**
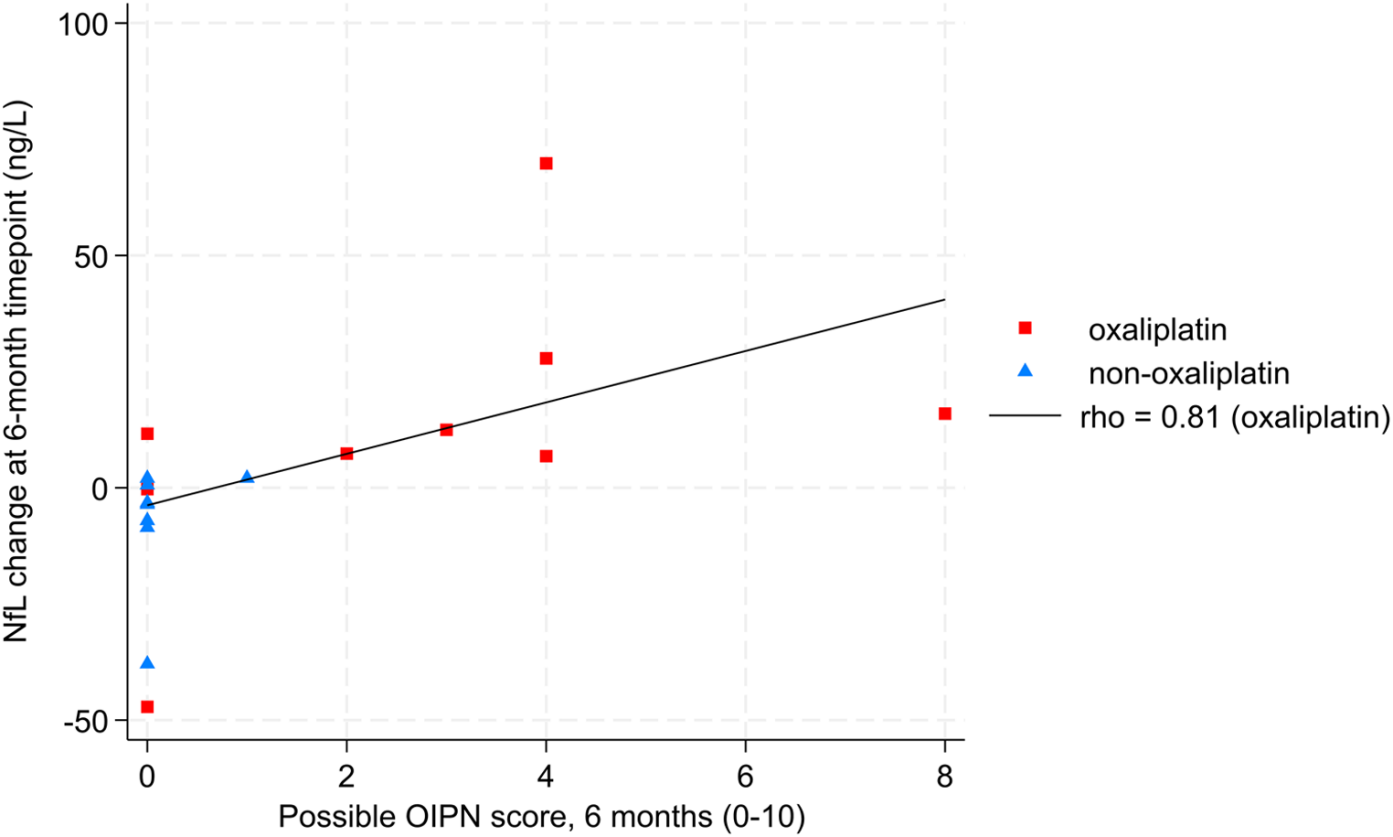
Scatterplot of possible OIPN score against changes in neurofilament light chain levels at the 6-month-timepoint compared to baseline (delta NfL). The black line indicates the best-fitted line for NfL measurements from the oxaliplatin group. *N* = 9 (oxaliplatin)

Spearman correlation of Δ3NfL levels and possible OIPN scores at 3 months showed a moderate but not significant correlation (rho 0.48 *p* = 0.08) and a strong significant correlation between Δ6NfL and possible OIPN score at 6 months (rho 0.81, *p* < 0.001) in the oxaliplatin group. The Δ6NfL and possible OIPN score correlation is visualized in Fig. 7.

#### Cumulative oxaliplatin dosage correlated to NfL and possible OIPN score at 3 and 6 months

Median cumulative oxaliplatin dose at 3 months was 468 mg/m^2^ (IQ: 385–518) and 494 mg/m^2^ (IQR: 385–814) at 6 months. Linear regression analysis adjusted for age to predict NfL concentrations in relation to accumulated oxaliplatin dosage was − 0.05 (95%CI: − 0.09– − 0.01, *p* = 0.02) per mg/m^2^ cumulated oxaliplatin at 3 months and 0.005 ng/L (95%CI-0.06-0.07, *p* = 0.16) at 6 months. Unadjusted and diabetes-adjusted linear regression analyses were nonsignificant (suppl. Table S2). Linear regression analyses of possible OIPN scores in relation to accumulated oxaliplatin were not significant (suppl. Table S2).

## Discussion

We studied the changes in NfL concentration during oxaliplatin treatment using a human cell model and a clinical study including patients with colorectal cancer. Our in vitro data showed concentration-dependent morphological changes in iPSC-derived sensory neurons (iPSC-SNs) when exposed to oxaliplatin. Additionally, clinically relevant concentrations of oxaliplatin led to measurable increased NfL concentrations in the medium from iPSC-SNs. Our clinical study supported these findings, with serum NfL levels at 3 and 6 months significantly elevated in patients receiving oxaliplatin compared to the control group. We observed a tendency towards a correlation between changes in NfL concentrations and possible OIPN scores at 3 and 6 months. However, the result was exploratory due to sample size, missing values, and considerable individual variation.

Prior research with iPSC-derived models suggests that platinum-based neurotoxicity observed as morphological changes occur only after longer exposure times and concentrations higher than those found in clinical settings [22]. In our study, oxaliplatin concentrations were selected based on clinical pharmacokinetic data and adjusted to account for the absence of repeated oxaliplatin exposure, making the observed increased NfL release from an oxaliplatin concentration of 10 µM and up relevant.

Consistently, recent studies report NfL release from IPSC-SNs following axonal damage caused by paclitaxel, indicating that NfL might be released after exposure to various neurotoxic chemotherapies [15, 17]. The effect of oxaliplatin on the morphology of sensory neurons, as shown in Fig. 1, indicates a reduction of the axonal neuronal network, likely caused by the clustering of many small axons into larger ones. These morphological changes differ from the axonal retraction seen after paclitaxel exposure and the axon fragmentation and abolition observed following vincristine exposure [25].

The iPSC-SN model has been established as a robust and reliable method to study CIPN and evaluate potential biomarkers on a cellular level [26, 27]. NfL measurement following oxaliplatin exposure has not been explored previously and represents a promising area for future in vitro studies. However, as observed in this study, donor variability remains a well-recognized challenge, stemming from genetic and non-genetic factors or differences in culturing and maintenance [28]. To mitigate some of these variations, iPSC-SNs derived from patients with known high or low susceptibility to oxaliplatin and OIPN would be valuable. Nonetheless, the observed donor variation in vitro underscores the existence of interindividual differences, which are also evident in the clinical presentation of OIPN, ranging from an absence of symptoms to debilitating neuropathy after a single oxaliplatin cycle. Additionally, we acknowledge the limitations of the in vitro model in general, particularly its lack of supportive cells such as glial cells. These cells and the surrounding microenvironment influence OIPN and NfL release development and modulation [20].

The prevalence of possible OIPN, dose reduction, and early cessation of oxaliplatin treatment align with findings in other studies [3, 29]. The definition of possible OIPN has previously been used and is based on the most common symptoms of OIPN [1]. However, the grading of possible OIPN has not been validated and is a coarse scale. The missing correlation between this grading scale and cumulated oxaliplatin, which is an established fact, stresses this point. The prefix “possible” reflects the grading tool’s reliance on interview-based symptom descriptions, lacking clinical signs and objective assessments which would increase the certainty of the OIPN diagnosis.

Two other studies have investigated the clinical correlation between NfL and OIPN, reporting results similar to those observed in our research [29, 30]. Comparable conclusions have also been made with CIPN caused by paclitaxel, although NfL concentrations reach higher levels during paclitaxel treatment [15, 16, 18]. One reason why oxaliplatin does not provoke similar changes in NfL compared to paclitaxel may be the suggested different mechanism of OIPN and paclitaxel-induced peripheral neuropathy, respectively, in which paclitaxel is suggested to cause more direct damage to axonal nerves than oxaliplatin [31].

NfL is released due to axonal damage in the CNS and the peripheral nervous system (PNS), which warrants caution when evaluating its potential as a biomarker. Confounding conditions from both PNS and CNS may influence the levels of NfL. This could, in part, explain the significant interindividual variation in NfL levels seen in this clinical study. In some CNS conditions NfL as a biomarker has shown its worth [14]. However, in PNS conditions such as diabetic polyneuropathy, despite the phenotypic similarities to CIPN, the evidence is debatable [32, 33]. Additionally, mechanisms regarding NfL and its release to the cerebrospinal fluid and subsequent blood, its degradation, turnover and clearance are still to be fully elucidated [11]. Only one study has studied the half-life of NfL in this group of patients [17]. Although unrelated to neuronal toxicity, these mechanisms can affect the measurable NfL levels. This underlines the need for extra awareness of potential confounders and elements to adjust for when evaluating NfL levels in relation to CIPN.

For NfL to function as an early biomarker of CIPN and aid in clinical decisions regarding dose reduction and premature cessation of oxaliplatin, NfL concentrations after one or two cycles should predict CIPN. Studies by Kateri et al. and Mortensen et al. found correlations between early increases in NfL and paclitaxel-induced peripheral neuropathy at treatment end [16, 17], indicating the potential of NfL as an early biomarker of taxane-induced peripheral neuropathy. However, Velasco et al. found a delay in NfL increased correlation to administered oxaliplatin cycles which may affect the utility of NfL as an early biomarker of OIPN. The sample size was equally small as in our study [29]. Therefore, future studies on OIPN and NfL need to be of a larger sample size and should have blood samples relevantly correlated to chemotherapeutic treatment along with objective evaluation of CIPN with validated examinations, scoring systems, and patient-reported outcome data. We have initiated project OxaNeuro with the abovementioned setup [34].

The main strength of our study is the combination of in vitro and clinical data. Furthermore, we had a homogenous patient group with the same diagnosis, and we included a control group to study NfL levels in cancer patients who did not receive oxaliplatin. A limitation of our in vitro study is that only a single exposure of oxaliplatin was assessed, as it was impossible to carry out multiple oxaliplatin exposures in the cell culture. In patients, OIPN is often a result of cumulative exposure. The oxaliplatin dose and exposure time were increased to compensate, but it would be valuable to investigate the effect of repeated oxaliplatin exposure in iPSC-SNs. Additionally, neuronal damage to the iPSC-SNs was observed but not quantified as it was outside this project’s scope, but it would have been valuable. The main limitations of the clinical setup are the low number of patients and the high number of missing data rendering the results exploratory. Unfortunately, we did not have any tests for neuropathy, i.e. no clinical examination and not any of the two gold-standard tests for confirmed neuropathy: nerve conduction studies and skin biopsy for intraepidermal nerve fiber density evaluation. We could therefore only reach the diagnosis of possible OIPN. Furthermore, the lack of relevant timing between oxaliplatin exposure and NfL measurements, together with an OIPN grading tool that is not validated, adds to the limitations.

In summary, we provide in vitro and clinical evidence of NfL release upon oxaliplatin exposure. We also found a correlation between increasing NfL concentrations and symptoms of OIPN in the clinical study, though most patients showed only a small increase. Prospective studies are needed to further evaluate NfL as an early biomarker for OIPN, with particular focus on the timing of blood sampling during chemotherapy and optimizing the clinical use of NfL to enable timely reduction of oxaliplatin, thereby preventing long-term OIPN in patients.

## Electronic supplementary material

Below is the link to the electronic supplementary material.

Supplementary Material 1

Supplementary Material 2

## Data Availability

All data supporting the findings in the invitro study are available within the paper and its Supplementary Information. The data that support the clinical findings of this study are not openly available due to reasons of sensitivity and are available from the corresponding author upon reasonable request and acceptance from the Ethical Committee of the Southern region of Denmark.

## References

[1] Bennedsgaard K, Ventzel L, Themistocleous AC, Bennett DL, Jensen AB, Jensen AR et al (2020) Long-term symptoms of polyneuropathy in breast and colorectal cancer patients treated with and without adjuvant chemotherapy. April 5114–5123. 10.1002/cam4.3129 10.1002/cam4.3129 PMC7367625 32469145

[2] Grothey A (2005) Clinical management of oxaliplatin-associated neurotoxicity. Clin Colorectal Cancer 5(Suppl 1):S38–46. 10.3816/ccc.2005.s.006 15871765 10.3816/ccc.2005.s.006

[3] Ventzel L, Jensen AB, Jensen AR, Jensen TS, Finnerup NB (2016) Chemotherapy-induced pain and neuropathy: a prospective study in patients treated with adjuvant oxaliplatin or docetaxel. Pain 157(3):560–568. 10.1097/j.pain.0000000000000404 26529271 10.1097/j.pain.0000000000000404

[4] Mols F, Beijers T, Vreugdenhil G (2014) Chemotherapy-induced peripheral neuropathy and its association with quality of life: a systematic review. 2261–2269. 10.1007/s00520-014-2255-7 10.1007/s00520-014-2255-7 24789421

[5] Kerckhove N, Collin A, Conde S, Chaleteix C, Pezet D, Balayssac D (2017) Long-Term effects, pathophysiological mechanisms, and risk factors of Chemotherapy-Induced peripheral neuropathies: A comprehensive literature review. Front Pharmacol 8:86. 10.3389/fphar.2017.00086 28286483 10.3389/fphar.2017.00086 PMC5323411

[6] Cavaletti G, D’Acunti A, Porcu A, Masiello G, Del Campo L, Traclò G et al (2023) Self-Reported assessment of the Socio-Economic impact of anticancer Chemotherapy-Related neurotoxicity. Toxics 11(2). 10.3390/toxics11020104 10.3390/toxics11020104 PMC9966709 36850979

[7] Shah A, Hoffman EM, Mauermann ML, Loprinzi CL, Windebank AJ, Klein CJ et al (2018) Incidence and disease burden of chemotherapy-induced peripheral neuropathy in a population-based cohort. J Neurol Neurosurg Psychiatry 89(6):636–641. 10.1136/jnnp-2017-317215 29439162 10.1136/jnnp-2017-317215 PMC5970026

[8] Pike CT, Birnbaum HG, Muehlenbein CE, Pohl GM, Natale RB (2012) Healthcare costs and Workloss burden of patients with chemotherapy-associated peripheral neuropathy in breast, ovarian, head and neck, and nonsmall cell lung cancer. Chemother Res Pract 2012:913848. 10.1155/2012/913848 22482054 10.1155/2012/913848 PMC3312207

[9] Michalova Z, Szekiova E, Blasko J, Vanicky I (2023) Prevention and therapy of chemotherapy-induced peripheral neuropathy: a review of recent findings. Neoplasma 70(1):15–35. 10.4149/neo_2022_221007N992 36573482 10.4149/neo_2022_221007N992

[10] Smith EML, Pang H, Cirrincione C, Fleishman S, Paskett ED, Ahles T et al (2013) Effect of Duloxetine on pain, function, and quality of life among patients with Chemotherapy-Induced painful peripheral neuropathy. JAMA 309(13). 10.1001/jama.2013.2813 10.1001/jama.2013.2813 PMC3912515 23549581

[11] Gafson AR, Barthelemy NR, Bomont P, Carare RO, Durham HD, Julien JP et al (2020) Neurofilaments: Neurobiological foundations for biomarker applications. Brain 143(7):1975–1998. 10.1093/brain/awaa098 32408345 10.1093/brain/awaa098 PMC7363489

[12] Wilson DH, Rissin DM, Kan CW, Fournier DR, Piech T, Campbell TG et al (2016) The Simoa HD-1 analyzer: A novel fully automated digital immunoassay analyzer with Single-Molecule sensitivity and multiplexing. J Lab Autom 21(4):533–547. 10.1177/2211068215589580 26077162 10.1177/2211068215589580

[13] Meregalli C, Fumagalli G, Alberti P, Canta A, Chiorazzi A, Monza L et al (2020) Neurofilament light chain: a specific serum biomarker of axonal damage severity in rat models of Chemotherapy-Induced peripheral neurotoxicity. Arch Toxicol 94(7):2517–2522. 10.1007/s00204-020-02755-w 32333051 10.1007/s00204-020-02755-w

[14] Khalil M, Teunissen CE, Otto M, Piehl F, Sormani MP, Gattringer T et al (2018) Neurofilaments as biomarkers in neurological disorders. Nat Rev Neurol 14(10):577–589. 10.1038/s41582-018-0058-z 30171200 10.1038/s41582-018-0058-z

[15] Huehnchen P, Schinke C, Bangemann N, Dordevic AD, Kern J, Maierhof SK et al (2022) Neurofilament proteins as a potential biomarker in chemotherapy-induced polyneuropathy. JCI Insight 7(6). 10.1172/jci.insight.154395 10.1172/jci.insight.154395 PMC8986065 35133982

[16] Karteri S, Bruna J, Argyriou AA, Mariotto S, Velasco R, Alemany M et al (2022) Prospectively assessing serum neurofilament light chain levels as a biomarker of paclitaxel-induced peripheral neurotoxicity in breast cancer patients. J Peripher Nerv Syst 27(2):166–174. 10.1111/jns.12493 35384143 10.1111/jns.12493

[17] Mortensen C, Steffensen KD, Simonsen E, Herskind K, Madsen JS, Olsen DA et al (2023) Neurofilament light chain as a biomarker of axonal damage in sensory neurons and paclitaxel-induced peripheral neuropathy in patients with ovarian cancer. Pain 164(7):1502–1511. 10.1097/j.pain.0000000000002840 36508173 10.1097/j.pain.0000000000002840

[18] Cavaletti G, Pizzamiglio C, Man A, Engber TM, Comi C, Wilbraham D (2023) Studies to assess the utility of serum neurofilament light chain as a biomarker in Chemotherapy-Induced peripheral neuropathy. Cancers (Basel) 15(17). 10.3390/cancers15174216 10.3390/cancers15174216 PMC10486738 37686492

[19] Cebulla N, Schirmer D, Runau E, Flamm L, Gommersbach S, Stengel H et al (2023) Neurofilament light chain levels indicate acute axonal damage under bortezomib treatment. J Neurol 270(6):2997–3007. 10.1007/s00415-023-11624-2 36802032 10.1007/s00415-023-11624-2 PMC10188420

[20] Kang L, Tian Y, Xu S, Chen H (2021) Oxaliplatin-induced peripheral neuropathy: clinical features, mechanisms, prevention and treatment. J Neurol 268(9):3269–3282. 10.1007/s00415-020-09942-w 32474658 10.1007/s00415-020-09942-w

[21] Mortensen C, Thomsen MT, Chua KC, Hammer HS, Nielsen F, Potz O et al (2024) Modeling mechanisms of chemotherapy-induced peripheral neuropathy and chemotherapy transport using induced pluripotent stem cell-derived sensory neurons. Neuropharmacology 258:110062. 10.1016/j.neuropharm.2024.110062 38972371 10.1016/j.neuropharm.2024.110062

[22] Mortensen C, Andersen NE, Stage TB (2022) Bridging the translational gap in Chemotherapy-Induced peripheral neuropathy with iPSC-Based modeling. Cancers (Basel) 14(16). 10.3390/cancers14163939 10.3390/cancers14163939 PMC9406154 36010931

[23] Digitale JC, Martin JN, Glymour MM (2022) Tutorial on directed acyclic graphs. J Clin Epidemiol 142:264–267. 10.1016/j.jclinepi.2021.08.001 34371103 10.1016/j.jclinepi.2021.08.001 PMC8821727

[24] Vinter C, Hviid B, Knudsen CS, Parkner T (2020) Reference interval and preanalytical properties of serum neurofilament light chain in Scandinavian adults. Scand J Clin Lab Investig 80(4):291–295. 10.1080/00365513.2020.1730434 32077769 10.1080/00365513.2020.1730434

[25] Mortensen C, Chua KC, Hammer HS, Nielsen F, Pötz O, Svenningsen ÅF et al Paclitaxel- and vincristine-induced neurotoxicity and drug transport in sensory neurons. BioRxiv. 2023:2023.02.07.527432. 10.1101/2023.02.07.527432

[26] Xiong C, Chua KC, Stage TB, Priotti J, Kim J, Altman-Merino A et al (2021) Human induced pluripotent stem cell derived sensory neurons are sensitive to the neurotoxic effects of Paclitaxel. Clin Transl Sci 14(2):568–581. 10.1111/cts.12912 33340242 10.1111/cts.12912 PMC7993321

[27] Schinke C, Fernandez Vallone V, Ivanov A, Peng Y, Kortvelyessy P, Nolte L et al (2021) Modeling chemotherapy induced neurotoxicity with human induced pluripotent stem cell (iPSC) -derived sensory neurons. Neurobiol Dis 155:105391. 10.1016/j.nbd.2021.105391 33984509 10.1016/j.nbd.2021.105391

[28] Volpato V, Webber C (2020) Addressing variability in iPSC-derived models of human disease: guidelines to promote reproducibility. Dis Model Mech 13(1). 10.1242/dmm.042317 10.1242/dmm.042317 PMC6994963 31953356

[29] Velasco R, Marco C, Domingo-Domenech E, Stradella A, Santos C, Laquente B et al (2024) Plasma neurofilament light chain levels in chemotherapy-induced peripheral neurotoxicity according to type of anticancer drug. Eur J Neurol e16369. 10.1111/ene.16369 10.1111/ene.16369 PMC11295167 38952074

[30] Kim S-h, Choi MK, Park NY, Hyun J-w, Lee MY (2020) Serum neurofilament light chain levels as a biomarker of neuroaxonal injury and severity of oxaliplatin-induced peripheral neuropathy. Sci Rep 1–9. 10.1038/s41598-020-64511-5 10.1038/s41598-020-64511-5 PMC7224372 32409710

[31] Kacem H, Cimini A, d’Angelo M, Castelli V (2024) Molecular and cellular involvement in CIPN. Biomedicines 12(4). 10.3390/biomedicines12040751 10.3390/biomedicines12040751 PMC11048589 38672107

[32] Fundaun J, Kolski M, Molina-Alvarez M, Baskozos G, Schmid AB (2022) Types and concentrations of Blood-Based biomarkers in adults with peripheral neuropathies: A systematic review and Meta-analysis. JAMA Netw Open 5(12):e2248593. 10.1001/jamanetworkopen.2022.48593 36574244 10.1001/jamanetworkopen.2022.48593 PMC9857490

[33] Maatta LL, Andersen ST, Parkner T, Hviid CVB, Bjerg L, Kural MA et al (2023) Serum neurofilament light chain - A potential biomarker for polyneuropathy in type 2 diabetes? Diabetes Res Clin Pract 205:110988. 10.1016/j.diabres.2023.110988 38349953 10.1016/j.diabres.2023.110988

[34] Gehr NL, Karlsson P, Timm S, Christensen S, Hvid CA, Peric J et al (2024) Study protocol: fish oil supplement in prevention of oxaliplatin-induced peripheral neuropathy in adjuvant colorectal cancer patients - a randomized controlled trial. (OxaNeuro). BMC Cancer 24(1):168. 10.1186/s12885-024-11856-z 38308227 10.1186/s12885-024-11856-z PMC10837958

