# Supplementary material for: Neurofilament light chain as a marker for neuronal damage: integrating in vitro studies and clinical findings in patients with oxaliplatin-induced neuropathy
Source: Cancer Chemother Pharmacol. 2025 Apr 10;95(1):53. doi: 10.1007/s00280-025-04773-w (PMC11985616; doi:10.1007/s00280-025-04773-w)
Supplement: Supplementary file 1 — Supplementary Material 1 [file 280_2025_4773_MOESM1_ESM.docx]

**Title: Neurofilament light chain as a marker for neuronal damage: integrating in vitro studies and clinical findings in patients with oxaliplatin-induced neuropathy**

Journal: Cancer Chemotherapy & Pharmacology

Author: Nina Lykkegaard Gehr, Danish Pain Research Center, Department of Clinical Medicine, Aarhus University, Denmark, [ninalykgehr@clin.au.dk](mailto:ninalykgehr@clin.au.dk)

**Supplementary Table S1**

**Absolute NfL levels and protein concentrations of iPSC-derived sensory neurons exposed to control (0.2% sterile water) or indicated concentrations of oxaliplatin for 96 hours. NfL levels were measured using single-molecule array, and protein concentrations were determined by bicinchoninic acid (BCA) assay. The experiments were performed with iPSC donors, A18945 and WTC-11.**

| Sample ID | Condition | Donor | NfL (pg/mL) | Protein (µg/mL) |
| --- | --- | --- | --- | --- |
| CM029/1 | Control | A18945 | 100422 | 673,14 |
| CM029/2 | Control | A18945 | 148417 | 786,92 |
| CM029/3 | Control | A18945 | 117968 | 732,01 |
| CM029/4 | 1 µM oxaliplatin | A18945 | 122447 | 653,42 |
| CM029/5 | 1 µM oxaliplatin | A18945 | 106925 | 656,47 |
| CM029/6 | 1 µM oxaliplatin | A18945 | 93089 | 677,75 |
| CM029/7 | 10 µM oxaliplatin | A18945 | 125921 | 667,89 |
| CM029/8 | 10 µM oxaliplatin | A18945 | 102910 | 606,14 |
| CM029/9 | 10 µM oxaliplatin | A18945 | 111873 | 582,39 |
| CM029/10 | 20 µM oxaliplatin | A18945 | 147630 | 525,08 |
| CM029/11 | 20 µM oxaliplatin | A18945 | 162458 | 600,56 |
| CM029/12 | 20 µM oxaliplatin | A18945 | 173076 | 602,19 |
| CM030/1 | Control | WTC-11 | 45953 | 455,79 |
| CM030/2 | Control | WTC-11 | 39983 | 456,41 |
| CM030/3 | Control | WTC-11 | 37351 | 441,36 |
| CM030/4 | 1 µM oxaliplatin | WTC-11 | 43400 | 421,13 |
| CM030/5 | 1 µM oxaliplatin | WTC-11 | 36448 | 476,38 |
| CM030/6 | 1 µM oxaliplatin | WTC-11 | 43768 | 618,03 |
| CM030/7 | 10 µM oxaliplatin | WTC-11 | 53774 | 443,87 |
| CM030/8 | 10 µM oxaliplatin | WTC-11 | 61118 | 418,67 |
| CM030/9 | 10 µM oxaliplatin | WTC-11 | 64457 | 390,82 |
| CM030/10 | 20 µM oxaliplatin | WTC-11 | 198202 | 326,03 |
| CM030/11 | 20 µM oxaliplatin | WTC-11 | 183618 | 281,44 |
| CM030/12 | 20 µM oxaliplatin | WTC-11 | 214081 | 305,00 |
