# Supplementary material for: Neurofilament light chain as a marker for neuronal damage: integrating in vitro studies and clinical findings in patients with oxaliplatin-induced neuropathy
Source: Cancer Chemother Pharmacol. 2025 Apr 10;95(1):53. doi: 10.1007/s00280-025-04773-w (PMC11985616; doi:10.1007/s00280-025-04773-w)
Supplement: Supplementary file 2 — Supplementary Material 2 [file 280_2025_4773_MOESM2_ESM.docx]

**Title: Neurofilament light chain as a marker for neuronal damage: integrating in vitro studies and clinical findings in patients with oxaliplatin-induced neuropathy**

Journal: Cancer Chemotherapy & Pharmacology

Author: Nina Lykkegaard Gehr, Danish Pain Research Center, Department of Clinical Medicine, Aarhus University, Denmark, [ninalykgehr@clin.au.dk](mailto:ninalykgehr@clin.au.dk)

**Supplementary Table S2**

**Linear regression analyses of neurofilament light chain protein (NfL) and a short version of the Possible OIPN score in relation to cumulative oxaliplatin dose at 3 and 6 months. Both unadjusted and adjusted for age and diabetes.**

|  | **No** | **Unadjusted** | **95% CI** | **p** | **SE** | **Adjusted for age** | **95%CI** | **p** | **SE** | **Adjusted for diabetes** | **95%CI** | **p** | **SE** |
| --- | --- | --- | --- | --- | --- | --- | --- | --- | --- | --- | --- | --- | --- |
| **NfL (ng/L)** | | | | | | | | | | | | | |
| **3 month** | 13 | -0.05 | -0.11 - 0.004 | 0.06 | 0.03 | -0.05 | -0.09- -0.01 | 0.02 | 0.018 | -0.05 | -0.11-0.01 | 0.08 | 0.03 |
| **6 month** | 13 | 0.01 | -0.05 - 0.07 | 0.74 | 0.03 | 0.005 | -0.06 -0.07 | 0.87 | 0.03 | 0.02 | -0.05-0.09 | 0.54 | 0.03 |
| **Possible OIPN score** | | | | | | | | | | | | | |
| **3 months** | 9 | -0.01 | -0.03 – 0.01 | 0.38 | 0.01 | -0.006 | -0.03 -0.01 | 0.49 | 0.01 | -0.01 | -0.04- 0.01 | 0.14 | 0.01 |
| **6 months** | 10 | 0.004 | -0.004 – 0.01 | 0.28 | 0.003 | 0.004 | -0.004-0.01 | 0.27 | 0.003 | 0.005 | -0.003- 0.01 | 0.17 | 0.003 |
